# Supplementary material for: From sequence to enzyme mechanism using multi-label machine learning
Source: BMC Bioinformatics. 2014 May 19;15:150. doi: 10.1186/1471-2105-15-150 (PMC4229970; doi:10.1186/1471-2105-15-150)
Supplement: Additional file 2 — Java code of ml2db. Additional file ml2db_code.tar.gz contains the Java source code to run the multi-label machine learning experiments and save the results to database. The code’s Javadoc is included. [file 1471-2105-15-150-S2.zip › additional file 2/ml2db/ml2db/doc/test/ml2db/mulan/mulanarff/package-tree.html]

test.ml2db.mulan.mulanarff Class Hierarchy


JavaScript is disabled on your browser.


- Overview
- Package
- Class
- Use
- Tree
- Deprecated
- Index
- Help

- Prev
- Next

- Frames
- No Frames

- All Classes

# Hierarchy For Package test.ml2db.mulan.mulanarff

Package Hierarchies:

- All Packages

## Class Hierarchy

- java.lang.Object
  - junit.framework.Assert
    - junit.framework.TestCase (implements junit.framework.Test)
      - test.ml2db.mulan.mulanarff.IdInstanceTest
      - test.ml2db.mulan.mulanarff.IdMultiLabelInstancesTest
      - test.ml2db.mulan.mulanarff.MulanArffTest
      - test.ml2db.mulan.mulanarff.MulanDataSetDbLoaderTest
      - test.ml2db.mulan.mulanarff.MulanDataSetGeneratorTest
      - test.ml2db.mulan.mulanarff.MulanDataSetManagerTest
      - test.ml2db.mulan.mulanarff.MulanDataSetWriterTest
      - test.ml2db.mulan.mulanarff.MulanInstancesFillerTest
      - test.ml2db.mulan.mulanarff.SerializerTest
  - test.ml2db.mulan.mulanarff.StatisticsTest

- Overview
- Package
- Class
- Use
- Tree
- Deprecated
- Index
- Help

- Prev
- Next

- Frames
- No Frames

- All Classes
